# Repurposing Licensed Drugs for Use Against Alzheimer’s Disease

**DOI:** 10.3233/JAD-210080

**Published:** 2021-06-01

**Authors:** Leslie C. Norins

**Affiliations:** Alzheimer’s Germ Quest, Naples, FL, USA

**Keywords:** Alzheimer’s disease, amyloid-β, amyloid-β protein precursor, cognitive dysfunction

## Abstract

Substantial evidence, composed of drug mechanisms of action, *in vivo* testing, and epidemiological data, exists to support clinical testing of FDA-approved drugs for repurposing to the treatment of Alzheimer’s disease (AD). Licensed compound investigation can often proceed at a faster and more cost-effective manner than un-approved compounds moving through the drug pipeline. As the prevalence of AD increases with life expectancy, the current rise in life expectancy amalgamated with the lack of an effective drug for the treatment of AD unnecessarily burdens our medical system and is an urgent public health concern. The unfounded reluctance to examine repurposing existing drugs for possible AD therapy further impedes the possibility of improving the quality of patient lives with a terminal disease. This review summarizes some evidence which exists to suggest certain already-approved drugs may be considered for the treatment of AD and will perhaps encourage physicians to off-label prescribe these safe therapeutics.

## INTRODUCTION

Many therapeutic areas including cancer, erectile dysfunction, irritable bowel syndrome, and attention deficit disorder have benefited from repositioning existing drugs. The established safety and tolerability of approved therapeutics considered for repurposing can lower the burdensome financial thresholds associated with *in vitro* and *in vivo* screening, dose optimization, toxicology, formulation, and manufacturing development. Commencing clinical trials to establish a drug’s efficacy for the treatment of another disease is thus more accessible for pharmaceutical companies [1].

Although the underlying neurobiology and biochemistry of the orchestrated signaling cascades that definitively lead to the development of Alzheimer’s disease (AD) is still under study and a subject of scientific debate, a more immediate course of action that may provide symptomatic treatment and perhaps even disease-modifying therapies for AD, is the repurposing of FDA-approved substances [2]. These drugs may prove to function more effectively than the existing pharmacotherapies indicated for the treatment of AD, most of which only provide symptomatic relief for a six-month period [3]. Many of the drugs proposed here for repurposing possess a robust breadth of evidence for their effectiveness, while others require further investigation and further validation in standardized trials.

To facilitate the selection of approved drugs for further characterization against AD, this review summarizes some of the existing preclinical, epidemio-logical, and clinical evidence for top drug candidates, presents evidence against further pursuing the repurposing of some previously suggested drugs, and highlights other drugs which may be useful to assess in future randomized clinical trials. While acknowledging that there is currently no known animal model which exactly recapitulates human AD, and that often, this preclinical evidence does not translate to the bedside, this review delves into some of the *in vitro* and *in vivo* data that exist to support certain therapeutic candidates on the basis of improving AD-associated pathologies, functional disturbances and biomarkers, such as neuronal cell death, neuronal plasticity, Aβ deposition, and tau protein hyperphosphorylation. The therapies reviewed here represent a sample of convenience as they are the most prevalent therapeutics in the literature and have also been cited in several systematic reviews. Some of the reviewed therapeutics may have the potential to not only provide symptomatic relief, but perhaps even modify the disease state, in turn easing some of the social and economic burdens associated with AD.

Summarized evidence is presented for FDA-app-roved substances, with the potential to treat AD, in-cluding calcium channel blockers, phosphodiesterase inhibitors, insulin and glucagon-like peptide-1 (GLP-1) receptor agonists, non-steroidal anti-inflammatory drugs, antibiotics, stimulants, mood stabilizers, anti-virals, and antioxidants. The span of pharmacological categories covered serves to highlight the multif-arious effects of AD and suggests that one pharmacotherapy may be insufficient to combat this baffling disease.

## CALCIUM CHANNEL BLOCKERS

### Dihydropyridines

Although a correlation exists between hypertension and AD, hypertension often occurs along other vascular risk factors that have also been implicated in the progression of AD [4]. Calcium channel blockers (CCBs) provide vasodilatory effects on smooth muscle vasculature accounting for their benefit as antihypertensives. Easily crossing the blood-brain barrier to also increase blood flow to the brain, CCBs have also been suspected to grant neuroprotection as some evidence exists to indicate their potential to reduce the incidence of AD. Dihydropyridines such as nimodipine (FDA-approved for reducing the severity of ischemia), nivaldipine, and nitrendipine are some of the most widely available calcium channel blockers.

*In vitro* studies of CCBs have demonstrated their effectiveness in improving cell survival in presence of Aβ, rescuing Aβ-induced neurotoxicity, and dec-reasing overall Aβ production and oligomeric accumulation [5–7]. Other *in vitro* studies have indicated the protective effects of nimodipine in Aβ-induced cytotoxicity. Nimodipine reduced secretion of Aβ in other cell types of the brain such as in microglia [8].

*In vivo* studies further substantiate the use of dih-ydropyridine CCBs as treatment for AD. Not only have some *in vivo* studies on dihydropyridines such as nivaldipine significantly increased Aβ clearance in transgenic mouse models of AD, Tg APPsw (Tg2576) and Tg PS1/APPsw, but have also reversed memory and learning deficits measured through behavioral testing using the Morris Water Maze [9]. Indeed, other studies demonstrated the neuroprotective effects of nilvadipine, which prevented im-pairment of spatial memory and apoptosis in the hippocampus in rats stereotactically injected with Aβ—the circuit of the brain primarily responsible for learning by encoding declarative and spatial memories [10]. Likewise, nimodipine mitigated prevalent pathologies associated with AD, such as apopto-sis and pathological lesions in neurons of the hippocampus and cortex and inhibited tau hyperphosphorylation in rats with chronic cerebral hyperfusion (CCH) which promotes hyperphosphorylation of tau proteins [11]. These studies also indicated that nimodipine rescued spatial memory deficits induced by CCH.

Although substantial preclinical evidence exists to suggest that some dihydropyridines could treat AD in patients, the NILVAD study, involving participants over the age of 50 meeting NINCDS-ADRDA standards for diagnosis of probable AD, failed to show any cognitive benefit of treatment with nilvadipine [12]. However, the neuroprotective effects of nilvadipine on patients without diagnosed AD have not been tested. It is conceivable that a drug may only work in earlier disease phases but may fail to act once the disease is more progressed. Indeed, this study showed that patients at an earlier stage of AD in the experimental group performed better in memory and language measurements than the placebo group, with a∼50%decrease in cognitive decline. Further clinical studies are required to test the neuroprotective effects of nilvadipine on patients with a predisposition to AD without any clinical presentations of the disease. However, although nilvadipine is approved for use in Europe and Japan, it is not FDA-approved. Efforts should focus on other dihydropyridines that have gone through the rigorous approval process in the United States.

The similar chemical structure and proposed mechanisms of action of nimodipine to that of nilvadipine would suggest that nimodipine could also have no clinical benefits for patients with AD. A systematic review analyzed the efficacy of nimodipine on symptoms of dementia in individuals with AD, cerebrovascular disease, mixed AD, and cerebrovascular disease and in unclassified disease [13]. It encompassed 14 randomized clinical trials and surprisingly found an improvement in SCAG scale and cognitive function associated with the use of this drug. Although clinical trials have reported the efficacy of nimodipine in improving cognition, they were small, short, and did not directly measure treatment’s effect on progressive AD pathology. These limited, yet promising, clinical studies, as well as longitudinal epidemiological evidence suggesting the potential neuroprotective effects of dihydropyridine CCBs, can serve as preliminary rationale for a more robust clinical trial studying the effects of already-approved nimodipine on AD [14]. Unfortunately, there are currently no registered clinical trials on ClinicalTrials.gov nor the International Standard Randomized Controlled Trial Number (ISRCTN) databases.

### Dantrolene

Dantrolene is indicated for treatment of muscle spasticity. It is a CCB as it antagonizes ryanodine receptors (RyRs), thus inhibiting the release of Ca^2 +^ from endoplasmic reticulum (ER) stores. RyRs are increased in AD in the hippocampus, and excess Ca^2 +^ release from ER can lead to mitochondrial free radical release resulting in oxidative stress and neuronal cell death [15, 16]. Thus, it seems logical to consider dantrolene for the treatment of AD.

*In vitro* dantrolene increased the presence of antiapoptotic protein Bcl2 and decreased neuronal cell death [17]. Subsequent *in vivo* evidence from multiple studies implicates it as a potential therapeutic for AD. It reduced Aβ load in the hippocampus and memory deficits in both a Tg2576 and a triple transgenic AD mouse model (3xTg-AD), normalized ER Ca^2 +^ signaling and restored synaptic transmission and neuroplasticity (the molecular correlate of learning and memory) in 3xTg-AD mice [18–20]. As promising as these results are, no current registered clinical trial exists to test the effects of dantrolene on AD.

## PHOSPHODIESTERASE (PDE) INHIBITORS

### Sildenafil

Sildenafil, more commonly known by its brand name as Viagra, functions as a phosphodiesterase type 5 (PDE5) inhibitor and is used to treat erectile dysfunction and pulmonary hypertension. PDE5 protein is significantly upregulated in the temporal cortex of patients with AD [21]. Cyclic guanosine monophosphate (cGMP), degraded by PDE5, is present at lower concentrations in the cerebrospinal fluid (CFS) of patients with AD. Typically, cGMP upregulates expression of proliferator-activated rece-ptor-γ coactivator 1α (PGC1α)—thought to indire-ctly suppress Aβ generation [22, 23]. Thus, PDE5 has been proposed as a therapeutic target for AD.

*In vitro* sildenafil prevented the Aβ-induced oxi-dative stress and cell death [24]. Another study corroborated these findings by demonstrating decreased caspase activation and apoptosis after treatment with sildenafil in hippocampal cells [25]. *In vivo* studies demonstrated a memory improvement in rats with a concomitant increase in hippocampal cGMP levels after phosphodiesterase inhibitor administration [26]. Regular treatment with sildenafil also increased cognitive function and Aβ load in APP/PS1 AD mouse models, and mitigated tau pathology in the hippocampus of a senescence-accelerated mouse model (SAMP8) while ameliorating cognitive impairments [27, 28].

A link between cerebrovascular disease and AD pathology has been proposed [29]. Thus, improved cardiovascular function is a coveted goal for intervening with AD progression. To this end, a clinical study demonstrated the increase in cerebral oxygen after sildenafil administration in patients with AD [30]. Interestingly, a more recent pilot study revealed that sildenafil normalized fractional amplitude of low frequency fluctuations (fALff) in hippocampus (increased fALff has been revealed in AD) [31]. These and other results may be used as preliminary evidence to justify more clinical trials of this molecule against AD.

## INSULIN AND GLUCAGON-LIKE PEPTIDE-1 (GLP-1) RECEPTOR AGONISTS

### Nasal or infused insulin and liraglutide

Type 2 diabetes has been identified as a risk factor for AD due to the substantial overlap in comorbidities and potential pathomechanisms leading to each disease. It is characterized by impaired insulin signaling—imperative for glucose metabolism. Likewise, aberrant brain insulin signaling has been extensively documented in AD [32, 33]. Other than its involvement in bioenergetics and metabolism, the biochemical pathways which insulin regulates in the brain are still under study and are likely numerous. However, sizable evidence suggests a role for cerebral insulin in synaptic spine formation and viability, neurotransmitter turnover, inflammation, vasodilation, and more prominently the turnover of Aβ and tau phosphorylation [34]. Moreover, expression of glucose transporters at the blood-brain barrier is decreased even before the onset of AD pathological symptoms [35]. The putative multifarious pathways involving cerebral insulin suggest how dysregulation of insulin could be detrimental and potentially result in neurodegeneration. These rationales have led to studies of cerebral insulin and GLP-1 analogues as plausible treatments for AD [36].

GLP-1 analogues mimic glucagon-like peptide 1 which is a hormone that promotes the secretion of insulin, in turn lowering blood sugar. The well-esta-blished blood-brain barrier permeability of GLP-1 analogues such as liraglutide, make them great candidates for directly modifying neurobiology even when peripherally injected [37]. The FDA has approved Saxenda, Victoza (liraglutide), and Byetta (excendin-4) for use in the United States. These are liraglutide injectables indicated as adjuncts to exercise to promote glycemic control in individuals with Type 2 diabetes.

GLP-1 analogues *in vitro* demonstrated reduced cell death, AβPP and Aβ levels through mechanisms associated with glycogen synthase kinase 3β (GSK3β) and decreased tau phosphorylation [38, 39]. Similarly, insulin reduced intraneuronal Aβ [40].

*In vivo* mouse models have been used to demonstrate the neuroprotective effects of these analogues. Although Val(8)GLP-1 is a GLP-1 analogue specific for mice, it is important to point out that in APP/PS1 mice, Val(8)GLP-1 decreased load of Aβ plaques and protected synaptic plasticity [41]. Similarly, neuroplasticity was protected in type 2 diabetes mouse models (high-fat-diet-fed mice) injected with human GLP-1 analogue excendin-4 [42]. These mice also exhibited an increase in recognition index, indicating improve learning and memory.

The rate of neurogenesis, which normally occurs in brain regions such as the dentate gyrus (DG) of the hippocampus, is decreased as a result of AD. Another study in type 2 diabetes mouse models (leptin-deficient ob/ob, db/db, and high-fat-diet-fed mice) interestingly found neuronal progenitor cell proliferation in the DG of hippocampus in mice injected with liraglutide or excendin-4, suggesting an increase in neurogenesis [43]. Other *in vivo* studies in rats have also focused on the direct effects of insulin on inhibiting Aβ oligomers and restoring Aβ-induced suppression of neuroplasticity [44].

Clinical trials of GLP-1 analogues have demonstrated that liraglutide increased the blood-brain glucose transfer capacity in the cerebral cortex of subjects with AD [45]. As a result, cognition positively correlated with glucose utilization in study subjects. This is of particular importance since blood-brain transfer capacity diminishes with duration of AD. Currently there is another ongoing clinical trial studying liraglutide and its effects on AD [46]. The study is also looking at cerebral glucose metabolic rate, changes in cognitive and functional abilities and several other biomarkers; however, results are not yet available.

Aside from GLP-1 analogue administration, the direct delivery of insulin may have beneficial effects on patients with AD. In a pilot clinical study, nasal delivery of insulin improved delayed memory associated with mild cognitive impairment (MCI) or AD and preserved general cognition and changes in the cerebral metabolic rate of glucose in bilateral occipital, right temporal, bilateral frontal, and right precuneus and/or cuneus regions [47]. In exploratory analysis, improved cognition, in participants on insulin with MCI or AD, was associated with an increase in CSF Aβ_42_ levels and a decrease in tau protein/Aβ_42_ [47]. Another study involving intranasal insulin Detemir increased verbal working memory and visuospatial memory in adults with AD or MCI who were apolipoprotein E ɛ4 (*APOE* ɛ4) carriers [48]. Likewise, another study demonstrated that insulin improved memory in individuals diagnosed with MCI or AD who were treated with insulin, reduced the tau-P181/Aβ_42_ ratio and preserved or increased MRI volume in AD-associated brain regions (left cuneus, right middle cingulum, right parahippocampal gyrus, and left superior parietal cortex) [49]. Although the pre-clinical data, epidemiological studies, and some clinical data suggest a role for insulin in the treatment of AD, other randomized clinical studies have not found significant improvements in patients with AD who were administered infused or nasal insulin [50–52]. More studies are needed to detangle the conflicting results of these different trials and determine the effects of insulin on individuals with less advanced forms of AD. Perhaps individual characteristics determine whether a person responds or not.

## NON-STEROIDAL ANTI-INFLAMMATORY DRUGS (NSAIDS)

### Diclofenac

Increasing evidence suggests that inflammation is an early neuropathological event in AD [53, 54]. Diclofenac, like other NSAIDs, is currently approved for ameliorating pain and inflammation, and it is specifically indicated for reliving osteoarthritis and rheumatoid arthritis signs and symptoms. Preclinical research demonstrated that fenamate NSAIDs, chemically related to diclofenac, conferred neuroprotection in 3xTgAD mouse models [55].

Epidemiological evidence suggests a role for NSAIDs in reducing the risk of AD [56]. Specifically, a study analyzing the Alzheimer’s Disease Neuroimaging Initiative (ADNI) database used logistic regression and modeling to tease out prevalence of AD and cognitive decline for individuals taking commonly used NSAIDs. Paralleling animal studies, diclofenac use was negatively correlated with cognitive decline and AD incidence [57]. No other investigated NSAIDs had significant associations with cognitive abilities. Additionally, one recent obs-ervational cohort study found that AD frequency was significantly decreased in the diclofenac group compared to other NSAID groups [54]. This small, but promising result further bolsters the need for investigating the pharmacological interactions diclofenac participates in as regards AD.

## STIMULANTS

### Modafinil

Modafinil is a stimulant, pharmacologically distinct from other well-known ones, used to treat symptoms of excessive sleepiness caused by obstructive sleep apnea or narcolepsy [58]. It is thought to enhance cognitive performance—the most prevalent loss resulting from a neurodegenerative disease such as AD [59]. Additionally, modafinil improved hippocampal neurogenesis, global mental status, and attention [58].

Preclinical studies in both mice and rats show that not only does short-term treatment with modafinil promote DG hippocampal neurogenesis and decrease cell death, but also normalizes brain-derived neurotrophic factor (BDNF) expression, which is known to be deficient in individuals with AD [60, 61]. Other rat studies further demonstrated the behavioral benefits of modafinil on working memory as administration of it increased performance in the Morris Water Maze [62]. These studies did not test the effects of modafinil on AD animal models, which may explain why clinical studies have not been pursued more vigorously.

Clinical evidence for one study concluded that the administration of modafinil did not change apathy in individuals with AD [63]. However, no other AD-related symptomology was directly tested.

No registrations of clinical trials testing the effect of modafinil on AD currently exist.

## MOOD STABILIZERS

### Lithium

Even with well-established safety and tolerance data, and decades of use in the United States, a comprehensive list of lithium’s pharmacological me-chanisms have yet to be composed. However, even though lithium is currently indicated for the treatment of acute manic episodes, the element has been proposed to cover at least 16 biochemical pathways which become aberrant in AD [16]. The putative neuroprotective effects of lithium are thus too numerous to mention but include the regulation of oxidative stress, autophagy, mitochondrial dysfunction and inflammation (extensively reviewed in [16]).

Lithium significantly reduces tau phosphorylation and Aβ production by modulating AβPP processing in *in vivo* studies of AD mouse models (FTDP-17 tau and GSK-3β overexpressing mice) [64]. Likewise, micro-dosed lithium restored memory loss and hippocampal neurogenesis in AD-like amyloid pathology rats (McGill-R-Thy-APP transgenic rats) [65]. These same rats had reduced amyloid levels in the hippocampus. The attenuation of tau and Aβ pathology and the increased cognitive function shown in rodent models has given way to probing the effects of lithium on AD in humans.

Epidemiological data show a negative correlation between the prevalence of dementia and the lithium content in drinking water, suggesting that long-term exposure to lithium, even at microlevels, may prevent or reduce the severity of AD [66]. Indeed, a short clinical study reported the restoration of BDNF serum levels and increased cognitive improvement in patients with early AD [67]. Another clinical study reported increased memory and attention and decreased tau phosphorylation in the CSF of subjects taking lithium [68]. The continuation of this study concluded that lithium-treated subjects maintained cognitive stability for over two years after treatment [69]. It demonstrated an increase in Aβ in CFS of individuals, suggesting disease-modifying properties of lithium. The multitude of AD-associated pathways targeted by lithium indicate testing it for AD should be given high priority.

## ANTIVIRALS

### Acyclovir

Acyclovir is a nucleoside analog that acts as an antiviral compound used against herpes virus. Herpes is known to cause brain damage in several regions [70]. Given its direct impact on the brain, some in the scientific community postulate that herpes simplex virus 1 (HSV1) leads to the development of AD in *APOE* ɛ4 carriers by generating Aβ, precipitating hyperphosphorylation of tau and disrupting autophagy [71]. Plasma from 360 individuals was analyzed to reveal that heterozygous *APOE* ɛ4 subjects with antibodies for HSV1 had an increased risk for developing AD [72]. Other studies in postmortem tissue, showing the colocalization of HSV1 DNA to Aβ plaques, concur that HSV1 may be an etiological factor in AD [73].

A recent population-based cohort study concluded that anti-herpetic medication correlated with a decreased risk of developing dementia [74]. Although retrospective in nature and compiling data from patients taking other antivirals, the study also included data of patients taking acyclovir. Further evidence is needed to highlight causal effects of microbes on AD [75]. Additionally, more robust clinical trials on *APOE* ɛ4 carriers are needed to determine whether acyclovir, among other antivirals, is effective in preventing the onset of AD.

## ANTIBIOTICS

### Minocycline

Out of the many tetracycline antibiotics, minocycline is the most effective in crossing the blood-brain barrier. It is an anti-inflammatory drug with a broad spectrum of activity that serves as an antibiotic. Its antioxidant activity has also been shown to attenuate oxidative-stress induced neurotoxicity [76]. Minocycline also combats the effects of Aβ in pre-clinical studies and is known to address at least four known pathways which are aberrant in AD [16].

Minocycline stabilizes mitochondria and inhibits JNK activation [77]. JNK2 and JNK3 activation has been associated with plaques and neurofibrillary tangles [78]. Aβ-induced cytotoxicity and cleavage of AβPP was decreased by the inhibition of JNK, which led to a decrease of soluble Aβ oligomers [79]. *In vitro* evidence has demonstrated that minocycline not only inhibits aggregate formation of Aβ, but also destroys fibrils [80].

*In vivo* studies in high-fat-diet rats and 3xTg-AD mouse models reporting decreases in Aβ accumulation with concomitant behavioral improvements further position minocycline as a candidate for clinical trials [81, 82]. These reports included increased performance on spatial learning tasks and restored cortex-, hippocampus-, and amygdala-dependent learning and memory. Further preclinical studies in TG-SwDI transgenic mice demonstrated a reduction of pro-inflammatory markers after minocycline administration [83].

The promising *in vivo* results do not seem translatable. A clinical study found that patients with AD taking minocycline had no significant improvement compared to control subjects in Mini-Mental State Examination scores nor in Bristol Activities of Daily Living Scale scores [84]. However, these data are derived from a limited study where no biomarkers were used to confirm the patient’s AD diagnosis. The statistical power of the study also decreased when many participants dropped out of the study due to gastrointestinal and dermatological side-effects from the antibiotic. Further testing of cognitive and functional abilities and AD biomarker measurements is required to determine if minocycline is effective in treating AD. However, the feasibility of future studies is bleak given the high-dose requirements (400 mg) of the putative treatment.

### Rifamycin

Commonly used for the treatment of tuberculosis, rifamycin is another powerful antibiotic that has been suggested for the treatment of AD. *In vitro*, it reduced Aβ production while increasing Aβ clearance [85]. Further evidence exists to suggest rifamycin’s anti-amyloid, anti-inflammatory, anti-tau, and cholinergic effects [86].

Although preclinical *in vitro* studies suggest neuroprotective activity of rifamycin and even demonstrate pro-cognitive effects, limited clinical findings exist regarding the efficacy of this drug on AD [86]. However, rifampin, a semi-synthetic derivative of rifamycin, was used in conjunction with doxycycline to investigate their therapeutic role in the treatment of AD [87]. The antibiotic- treated groups experienced significantly less decline in the standardized Alzheimer’s Disease Assessment Scale cognitive subscale score. However, a more recent study reported contradictory findings by concluding that there was no significant benefit in cognition or function in AD patients receiving a dose of doxycycline and rifampin in combination or individually [88].

## ANTIOXIDANTS

### Melatonin

Aside from some of the above-mentioned substances which have antioxidant properties, but fit more cleanly into other pharmacological categories, melatonin is a powerful antioxidant [89]. It decreases with age, exposing the cells to increased oxidative stress, putatively contributing to neurodegenerative cascades which lead to AD. The increase in reactive oxygen species derived from aging could benefit from exogenous melatonin which may curb the associated increase in oxidized proteins and damaged DNA by scavenging free radicals. Melatonin is also believed to target inflammatory pathways associated with neurodegeneration.

It has been understood for over a decade that melatonin can prevent the formation of amyloid fibrils [90]. *In vitro* melatonin decreases secretion of soluble Aβ in different cell types and decreased the prevalence of proinflammatory cytokines such as IL-6 [91, 92]. Given its pleiotropic essence, melatonin also protects cells form apoptosis and oxidative damage conferred by Aβ-induction and attenuates tau hyperphosphorylation [93–95].

*In vivo* studies of Tg2576 transgenic mice revealed that melatonin further increased survival and inhibited oxidative pathology and amyloid deposition in brain regions such as cortex and [96, 97]. Additionally, melatonin mollified memory impairment, neu-roinflammation, and neurodegeneration in an aging mouse model [98]. These and other studies in rats injected with fibrillary Aβ revealed reduced level of reactive oxygen species and pro-inflammatory mediators [99]. In addition, melatonin decreased tau hyperphosphorylation, enhanced memory function and reduced oxidative stress in melatonin-biosyn-thesis-inhibited rats [100]. Interestingly, melatonin was more effective in mice before the first signs of hippocampal and cortical plaques compared to older mice that had already developed plaque deposition, an informative finding to guide recruitment for future clinical studies [96, 101].

Given these promising preclinical results, data on how melatonin impacts human subjects with dementia have been collected for several decades. It improved memory retention in older individuals [102]. In subjects with moderate to advanced dementia, sundowning was ameliorated by the use of melatonin [103]. Longer and more recent studies further shed light on its benefits to subjects with MCI, as these patients improved in cognitive examinations such as the Mattis’ test, Digit-symbol test, Trail A and B tasks, the Rey’s verbal test, Mini-Mental State Examination, and the AD Assessment Scale [104, 105]. Keeping in mind that these studies did not report any biomarker data associated with ameliorating disease but, given that melatonin is an over-the- counter medication, relatively inexpensive, and is also endogenously produced, patients with AD and *APOE* ɛ4 carriers could consider this substance as a low-risk potential co-treatment strategy.

## CONCLUSION

The heavy burden AD presents on our medical system is only increasing as life expectancy grows and the world’s population exponentially multiplies. Decreasing the severity or even onset of AD in the population, especially in predisposed individuals, by finding disease-modifying therapies might be achieved more quickly by repurposing medications for which clinical safety and tolerance has been well established. With potentially high benefit-risk ratios, re-establishing clinical trials for the purpose of studying the effects of approved drugs on AD can be the path of least resistance in the drug development pipeline.

This review has identified FDA-approved therapeutics, commonly cited in literature and systematic reviews, for repurposing as some evidence exists to suggest that they may be useful in the treatment of AD. Although this review focused on therapeutics commonly proposed in the literature, including systematic reviews, we did not perform a systematic review of our own and acknowledge that articles cited here represent a sample of convenience. Despite this limitation, which inevitably reduces the strength of our conclusion, we identified gaps of knowledge on this topic. Particularly we identified a lack of ongoing clinical trials to investigate repurposing existing therapeutics. We have also reviewed some issues with current evidence not translating from animal models to a clinical setting, and possible intolerability to patients of drugs like minocycline which are required at too high a dose. Further assessment of epidemiological data could help guide researchers in the choice of single or combination therapies to test in clinical trials. However, the most robust data to begin evidence-based clinical trials for priority candidate drugs as a treatment for AD may come from the combined efforts of preclinical research, epidemiological data, and in depth *in silico* transcriptional analyses, such as connectivity map (CMAP) project and platform-independent expression database (SPIED) [106]. The resulting top candidates have the potential to change the current, merely palliative, AD treatments into more proactive approaches.

Given the multitude of signaling pathways whose aberrant function is known to contribute to AD, there may not be a single drug that proves to be the panacea. A combination therapy addressing at least several, non-overlapping biochemical pathways of the 25 currently identified pathways may be the answer to treating and even modifying an AD state [16]. Fortuitously, some of the drugs described in this review, such as lithium, cover multiple pathways.

A combinatorial drug approach adds to the complexity of establishing clinical trials where multiple drugs are administered with no serious drug-drug interactions. Although establishing the safety of a combinatorial drug treatment sums another hurdle to establishing efficacy in the developmental pipeline, if we truly desire to curb the medical burden of this elusive disease, we must act promptly to enact clinical trials which address several questions. First, can a single drug or combination treatment *prevent* disease progression in patients with mild to moderate AD? Second, can a single drug or combination treatment *reverse* disease progression in patients with mild to advanced AD? Third, can a single drug or combination treatment *prevent* disease onset in individuals at increased risk of developing AD (*APOE* ɛ4 carriers)?

This third question arises from lessons that must be learned from failed trials and points that must be taken into consideration before proceeding with further clinical trials to repurpose drugs for AD treatment. First, the recruitment of elderly participants may confound the results of clinical trials as these participants suffer from comorbidities and other pathologies which make studying an already enigmatic-disease even more perplexing [107]. Second, some postulate the reason numerous clinical trials for AD therapies did not come to fruition was because the trials targeted a disease state that had already progressed beyond putative treatment or reversal. Although this is still an uncertain subject, it may be beneficial to identify the AD-prone population, such as *APOE* ɛ4 carriers, and commence AD treatment at initial stages of the disease, or even before any symptoms arise. Early detection is imperative since the degenerative process can commence 20–30 years before AD symptoms, during which time Aβ plaque deposition and neurofibrillary tangle amount [108].

Finally, because research exists in the real world and not an ivory tower, we must be cognizant of economic realities. The pharmaceutical industry is understandably loath to invest further hundreds of millions of dollars in field trials of compounds which cannot be patented and are generic. Thus, the two potential funders for such costly investigations are private foundations and the Unites States government. But how many researchers have proposed pertinent clinical trials to either?

## DISCLOSURE STATEMENT

The author’s disclosure is available online (https://www.j-alz.com/manuscript-disclosures/21-0080r2).
